# Pathophysiological Responses of Oral Keratinocytes After Exposure to Flavored E-Cigarette Liquids

**DOI:** 10.3390/dj13020060

**Published:** 2025-01-29

**Authors:** Abrar Shamim, Hannah Herzog, Raivat Shah, Sara Pecorelli, Virginia Nisbet, Ann George, Giancarlo A. Cuadra, Dominic L. Palazzolo

**Affiliations:** 1 Department of Biology, Muhlenberg College, 2400 W. Chew Street, Allentown, PA 18104, USA; ashamim@mgh.harvard.edu (A.S.); hannah.herzog@ucsf.edu (H.H.); raivat@med.umich.edu (R.S.); specorelli@muhlenberg.edu (S.P.); giancarlocuadra@muhlenberg.edu (G.A.C.); 2 Department of Oral and Maxillofacial Surgery, Massachusetts General Hospital, Boston, MA 02114, USA; 3 Department of Oral and Craniofacial Science, University of California San Francisco, 707 Parnassus Ave, San Francisco, CA 94143, USA; 4 University of Michigan Medical School, 1500 E. Medical Center Drive, Ann Arbor, MI 48109, USA; 5 DeBusk College of Osteopathic Medicine, Lincoln Memorial University, Harrogate, TN 37752, USA; vnisbet@uthsc.edu (V.N.); ann.george@sfhga.com (A.G.); 6 University of Tennessee Health Science Center, Nashville, TN 37205, USA; 7 St. Francis-Emory Healthcare, Columbus, GA 31904, USA

**Keywords:** ECIGs, oral epithelial cells, cytokines, glutathione, vaping, flavors, cellular morphology

## Abstract

Background: Electronic cigarettes (ECIGs) have grown in popularity, particularly among adolescents and young adults. Flavored ECIG-liquids (E-liquids) are aerosolized by these ECIGs and inhaled into the respiratory system. Several studies have shown detrimental effects of E-liquids in airway tissues, revealing that flavoring agents may be the most irritating component. However, research on the effects of E-liquids on biological processes of the oral cavity, which is the first site of aerosol contact, is limited. Hence, this study focuses on the effects of E-liquid flavors on oral epithelial cells using the OKF6/TERT-2 cell line model. Methodology: E-liquid was prepared with and without flavors (tobacco, menthol, cinnamon, and strawberry). OKF6/TERT-2 oral epithelial cells, cultured at 37 °C and 5% CO_2_, were exposed to 1% E-liquid ± flavors for 24 h. Outcomes determined include cell morphology, media pH, wound healing capability, oxidative stress, expression of mucin and tight junction genes, glycoprotein release, and levels of inflammatory cytokines (TNFα, IL-6, and IL-8). Results: Exposure to 1% flavored E-liquids negatively affect cellular confluency, adherence, and morphology. E-liquids ± flavors, particularly cinnamon, increase oxidative stress and production of IL-8, curtail wound healing recovery, and decrease glycoprotein release. Gene expression of *muc5b* is downregulated after exposure to E-liquids. In contrast, E-liquids upregulate *occludin* and *claudin-1*. Conclusions: This study suggests that ECIG use is not without risk. Flavored E-liquids, particularly cinnamon, result in pathophysiological responses of OKF6/TERT-2 cells. The dysregulation of inflammatory responses and cellular biology induced by E-liquids may contribute to various oral pathologies.

## 1. Introduction

Electronic cigarettes (ECIGs) were initially introduced in the United States in 2007 [1] as a tool for cessation of conventional tobacco use and as a less harmful means to satisfy nicotine addiction [2]; however, no consensus has been reached as to the possible adverse health effects of sustained ECIG usage [3,4]. Alarmingly, ECIGs have exponentially gained popularity in the last decade, notably in the adolescent population [5], paralleling the public health crisis concerning conventional cigarette use [6,7,8,9,10,11]. A 2015 United States Preventive Services Task Force report revealed conflicting evidence on the effectiveness of ECIGs as a means for smoking cessation [12]. Although advertised as a safer option, some recent studies suggest that ECIGs may be more harmful than initially predicted [13,14,15,16]. The lack of knowledge and longitudinal studies focusing on flavored ECIGs in the current context of increasing ECIG usage, particularly among adolescents, is a cause for major concern [17,18,19,20,21,22]. Consequently, the overall safety of ECIG usage must be further explored to determine potential long- and short-term health effects.

ECIG liquids (E-liquids) are typically vaporized by electronic nicotine delivery systems (ENDs), also referred to as ECIG devices. The E-liquid is usually composed of propylene glycol (PG), vegetable glycerin (VG), and variable concentrations of nicotine (0–24 mg/mL), and can be concocted with a wide range of flavoring agents [15,23]. In general, at concentrations less than 24 mg/mL, nicotine does not reduce viability in many human and mammalian cell lines [24,25,26,27,28], although there is evidence that nicotine can have pathological effects on HaCaT keratinocytes’ antimicrobial capabilities at concentrations as low as 8 mg/mL [29]. In contrast, cytotoxic effects have been reported with several flavors [30]. Tobacco and menthol flavors are commonly used by smokers who initiate ECIG usage for smoking cessation but are also trendy among adolescent populations [25,31,32,33,34]. Menthol, which has been associated with greater nicotine dependence [35], has been used as an ingredient in conventional cigarettes to make smoking initiation easier by reducing airway pain and irritation and suppressing coughing [36]. In addition, menthol is also a common flavoring agent for E-liquids. Fruit and sweet flavors play a role in attracting youth to ECIGs [31,37]. Candy and other dessert flavors are popular among the teenage population and correlate with a higher number of puffs per vaping session and a higher probability of vaping persistence after six months [20,38]. Cinnamaldehyde, the major component of cinnamon flavor, is involved with mechanisms of action associated with cytotoxicity [24,30,39,40,41], pro-inflammatory cytokines [39,40], reactive oxygen species [39,42], epithelial barrier dysfunction [43], and cytochrome c release leading to apoptosis [42].

ENDs heat and vaporize E-liquids, which the user inhales into the mouth and respiratory tract, thereby simulating conventional smoking in the absence of tobacco combustion. The oral cavity is the first anatomical site encountered by the ECIG vapors. During a vaping session, each inhalation coats the oral cavity and respiratory tract with the condensed E-liquid aerosol. The assimilation of the recondensed E-liquid with the mucous layer results in a wide range of physiological effects [44,45]. To date, the majority of E-liquid-related toxicity research is in regard to the lower respiratory tract, both in vivo and in vitro [23,46], namely concerning respiratory tissues such as bronchial epithelia [47,48,49,50,51,52]. In contrast, minimal research has been conducted on the effects of ECIG-generated aerosols on oral epithelial tissues. Among the limited studies on oral tissues, it has been reported that the saliva of ECIG users contains decreased levels of antimicrobial lysozyme and IgA in comparison to non-ECIG users [53], suggestive of a compromised immune response. Additionally, oral epithelial cells’ gene expression profiles and critical molecular pathways may be affected by unflavored aerosolized E-liquid treatments [54,55]. Adding flavorings to the E-liquid adds a layer of uncertainty, necessitating further research regarding their physiologic effects in the oral cavity. Sundar et al. 2016 report that some flavored E-liquid treatments led to oxidative stress and cytokine production in human periodontal ligament fibroblasts, human gingival epithelia, and epigingival 3D epithelia [56]. Additionally, some flavored E-liquids have been shown to increase DNA damage, inflammatory responses, and cytokine production in various oral epithelial cells [56,57,58,59]. ECIG use is implicated in negative impacts on the wound healing capacity of mucosal cells, both in vitro and in vivo, including complications in post-surgical wound healing [60]. The immune and oxidative responses of oral epithelial cells induced by ECIG flavorings must be further examined to contribute to the body of knowledge concerning their acute effects in the oral cavity.

Pathophysiological indicators in the oral mucosa include glutathione (GSH), cytokines, mucins, and tight junctions, among others. Reduced GSH, an intracellular antioxidant molecule, helps maintain the balance of reactive oxygen species (ROS) and combat oxidative injuries [61]. The sulfhydryl group on GSH permits its antioxidant capacity by neutralizing reactive oxygen species (ROS). In the presence of excess ROS, GSH is oxidized to form disulfides, either with itself to yield oxidized glutathione (GSSG), or with other sulfur-containing proteins or peptides [62]. In the event of oxidative stress, the total GSH (reduced and oxidized) increases, indicating oxidative stress [61,63]. In fact, the activity of γ-glutamyl cysteine synthase, the rate-limiting enzyme for the synthesis of GSH, has been shown to increase in response to oxidative stressors [64,65]. Alternatively, the amount of GSH could decrease as it is oxidized under exposure to oxidative stress [62], but this depletion of intracellular GSH usually signals apoptosis. For example, Ji et al. (2016) reported that unflavored aerosolized E-liquids may reduce intracellular GSH levels in normal human oral keratinocytes [66], suggestive of increased cytotoxic levels of oxidative stress [67]. Additionally, the inflammatory response of epithelial cells can be monitored by measuring the release of a number of pro-inflammatory cytokines such as Tumor Necrosis Factor (TNF) α, interleukin (IL)-6, and IL-8. These cytokines are signaling proteins that are under the control of transcriptional and post-transcriptional mechanisms in response to tissue injury [68] and are responsible for stimulating the large-scale host defense through acute phase responses, hematopoiesis, and a number of other immune reactions [68]. Elevated total glutathione represents a localized cellular oxidative challenge, while an increase in pro-inflammatory cytokines suggest the initiation of inflammation. An additional indicator of oral epithelial physiology that is investigated in this study is the expression of mucin glycoproteins, a critical component of mucosal function and salivary content [69], as well as tight junction genes, important in maintaining the epithelial and mucosal barrier function [70]. Specifically, mucin genes *muc1*, *muc4*, and *muc5b* and tight junction genes *occludin*, *claudin-1*, and *zonula occludens* (*ZO-1*) are investigated herein.

Oral epithelial cells play a critical role in wound closure post-injury. Wound closure helps to curtail the invasion of microbes and microbial products into the bloodstream and/or connective tissue [71]. A disruption in the ability of oral epithelial cells to heal following injury can pose a risk for infection and prolonged inflammation in addition to other systemic health risks [71]. A wound healing assay comparing the effects of E-liquid flavors on monolayer recovery following mechanical injury can provide insight into the flavor-dependent effects of ECIGs and is also demonstrated herein.

The aim of this study is to determine alterations in morphology, oxidative stress, gene expression of mucins and tight junctions, and wound recovery, as well as pro-inflammatory responses after exposure to E-liquids with and without flavors using the OKF6/TERT-2 cell model of oral keratinocytes. We predict that E-liquid flavorings will induce multifaceted physiologic disruptions in OKF6/TERT-2 cellular processes. Such disruptions could lead to adverse consequences in oral health, such as delayed/complicated post-surgical wound healing, development of periodontal disease, cariogenesis, and xerostomia, which ultimately could lead to systemic health effects including systemic inflammation, progression of cardiovascular disease, and poorer glycemic control in diabetics, among others [72].

## 2. Materials and Methods

### 2.1. Reagents and Supplies

Laboratory materials and reagents were obtained from Thermo Fisher Scientific (Waltham, MA, USA), unless otherwise noted.

### 2.2. Preparation of E-Liquids

Flavorless E-liquid was prepared by mixing food-grade PG and VG (Liquid Nicotine Wholesalers, Phoenix, AZ, USA) in a 1:1 *v*/*v* ratio and supplemented with 20 mg/mL (S)-(-)- nicotine (Alpha Aesar, Tewksbury, MA, USA), as previously described [30,73,74]. Four concentrated flavors (tobacco, menthol, cinnamon, and strawberry) in a primary diluent of PG were also purchased online from Liquid Nicotine Wholesalers (Phoenix, Arizona). According to Vapable.com, most manufacturers recommend “do it yourself” E-liquid recipes ranging from 5% to 25% flavors [75], depending on the specific flavor and user preference. With this in mind, for this study, flavored E-liquids were prepared by mixing each of the four concentrated flavors with flavorless E-liquid to yield a 5% flavored E-liquid mixture, representing the lowest end of the flavor range indicated above.

### 2.3. Preparation of Human Saliva

Saliva was collected from healthy volunteers under IRB approval code Cuadra_S19_18, as previously described [30,74,76]. In addition, verbal consent was obtained from all donors. Briefly, all volunteers were non-smokers, non-vapers, healthy at the time of donation, not on antibiotic treatments for at least 3 months, and had not consumed any foods or drinks (aside from water) within two hours prior to donation. Donated saliva was stored at −20 °C before processing. At the time of the experiments, saliva was thawed, pooled (thereby eliminating identification of individual samples) and reduced with 2.5 mM dithiothreitol (DTT), stirred on ice for 15 min. Reduced saliva was centrifuged at 14,000× *g* for 45 min to sediment any debris. The supernatant was collected, diluted 1:4 *v/v* with distilled water, and filter-sterilized through a 0.45 μm filter (VWR, Radnor, PA, USA). Sterile saliva was stored as 40 mL aliquots at −20 °C for up to a year. During use, saliva was kept at 4 °C for up to two weeks.

### 2.4. Preparation of Cell Culture Media

Keratinocyte Serum-Free Medium (KSFM) was prepared with 30 μg/mL of bovine pituitary extract (BPE), 3 ng/mL epithelial growth factor, 0.3 mM calcium chloride, 2 mM glutamine, and 100 U/mL penicillin and streptomycin. Dulbecco’s Modified Eagle’s Medium/Ham’s Nutrient Mixture F-12 (DMEM/F12) was supplemented with the same nutrients added to KSFM. A 1:1 *v*/*v* mixture of prepared DMEM/F12 and KSFM is from here on referred to as DFK [30]. Briefly the rationale for use of this media is two-fold: first, the cells grow confluent quickly, which saves time and effort; and second, DMEM/F12 is a more affordable reagent, which saves funds. We have previously reported any potential difference in gene expression between KSFM and DFK cultures, and found none [30].

### 2.5. Cell Cultures

OKF6/TERT-2 cells are oral mucosal epithelial cells from a human male that have been immortalized via telomerase 2 retroviral transduction and expression as well as deletion of p16INK4a regulatory protein [77]. This cell line was generated by the Rheinwald Lab at the Division of Dermatology, Department of Medicine and Harvard Skin Disease Research Center, Brigham and Women’s Hospital, Boston, MA, USA [77]. Cells were first cultured in 24-well flat-bottom plates with 1 mL of KSFM at 37 °C, 5% CO_2_ (standard conditions) overnight. The following day, cells were switched to DFK and cultured for three to four days. Once 90+% confluent, the cells were cultured with fresh DFK containing E-liquids ± flavors, including tobacco, menthol, cinnamon, and strawberry, or with hydrogen peroxide (serving as a positive control indicating toxicity) for 24 h at standard conditions.

### 2.6. OKF6/TERT-2 Cell Morphology and Supernatant pH

Confluent OKF6/TERT-2 cells were cultured in DFK containing 0.1, 0.5, or 1% E-liquids ± flavors, as described above. Twenty-four hours post-treatments, microscopic images of cell monolayers were taken using a Nikon Eclipse TE2000-U inverted microscope with a Nikon Digital Sight DS-Fi1 camera and NIS Elements Imaging Software (Nikon Instruments Inc, Melvin, NY, USA). All light microscopy images were captured at 100× magnification. It is worth noting that based on the results of this experiment (see the Section 3.1), where only 1% E-liquid ± flavors yield discernable visual differences, the remaining experiments were conducted using only the 1% E-liquid dose. Following microscopy, supernatants were stored at −20 °C for subsequent pH and SDS-PAGE analyses. 

### 2.7. Wound Healing Assay

A wound healing assay was conducted following the protocol previously described [30]. Briefly, OKF6/TERT-2 cells were cultured in 24-well plates until 90+% confluent. Using a pipet tip, a scratch was etched through the center of the wells. Cells were washed with 1 mL PBS and cultured in DFK with and without 1% E-liquids ± flavors under standard conditions. Monolayers with wounds were imaged as above at 0, 5, 10, 15, and 24 h post-injury. Quantitation of wound healing recovery rates was performed using the computer image processing program ImageJ, version 1.53t (National Institutes of Health), with the open source Wound Healing Size Tool plugin optimized for in vitro wound-heal assay analysis, as previously described [30,78,79]. The open wound gap size was defined by pixels^2^.

### 2.8. GSH Extraction

Pellets from 1% E-liquid-treated and control OKF6/TERT-2 cells were collected and stored at −80 °C until the time for GSH extraction. At the time of extraction, pellets were thawed at room temperature for approx. 30 min, followed by resuspension and lysing with 1 mL of 0.2% triton X-100. The mixtures were then vortexed for 3 min using a Disruptor Genie (Scientific Industries, Bohemia, NY, USA) to ensure complete cell lysis. The lysates were passed through a 0.22 µm filter using a 1 mL syringe, and the filtrates were aliquoted and stored at −20 °C until High Performance Liquid Chromatography (HPLC) analysis. An aliquot of the filtrate was set aside to determine protein content using a Pierce™ BCA Protein Assay Kit (Pierce Biotechnology, Thermo Fisher Scientific, Waltham, MA, USA) so that GSH could be expressed as nanomoles/mg protein.

### 2.9. HPLC Determinations of Total GSH

A Shimadzu HPLC system (Columbia, MD, USA) was used to quantify GSH and included the following: a spectrofluorometric detector (RF551), a pump (LC-20AT), a column oven (CTO-20A), an in-line membrane degasser (DGU-20A3R), and a Rheodyne 7725I manual injector with a 20 µL loop (30 µL injected). GSH in standards and samples was eluted from a Phenomenex (Torrance, CA, USA) 15 cm, Kinetex^®^ 5 µm reversed phase C-18 column. Column temperature was maintained at 37 °C. The HPLC methodology and the process of GSH derivatization with ortho-phthalaldehyde (OPA) is modified from Francioso et al. (2021) [80]. Briefly, GSH was tagged with OPA and detected fluorometrically using excitation and emission wavelengths set at 350 and 420, respectively. The mobile phase was delivered at a rate of 0.6 mL/minute in isocratic fashion and consisted of 25 mM Na_2_HPO_4_, pH 6.0. At the end of each day that samples were run, the mobile phase was refrigerated, and the column was inverted and flushed overnight with 100% methanol to remove accumulated protein. This step is necessary to prevent excess pressure increase on the system which leads to variable retention times. Chromatographic parameters were PC-controlled and analyzed using a Shimadzu Lab Solutions workstation (Columbia, MD, USA).

Reduced GSH, 98%, served as the standard. A stock solution (20 mM) of GSH was prepared in a 25 mM ammonium sulfate buffer containing 0.5% picric acid. From this stock GSH, working dilutions of 50, 25, 12.5, 6.25, and 3.125 µM were prepared in 0.2% triton X-100 (to simulate OKF6/TERT-2 lysed sample pellets; see above). Before HPLC analysis, 25 µL of standards or sample lysates were treated with 100 µL (1:5 *v*/*v*) of 10% tributylphosphine oxide solution in N, N-dimethylformamide (DMF) for the reduction of GSSG and other protein-bound GSH, consequently allowing for total GSH (both bound and unbound) determinations. The remaining free proteins were precipitated with 62.5 µL (1:2 *v*/*v*) with 10% metaphosphoric acid, followed by centrifugation (15,000× *g* for 30 min) at 4 °C. Twenty microliters of standard or sample supernatants were then mixed with 300 µL of 0.1 M phosphate buffer containing 0.1% ethylenediaminetetraacetic acid (EDTA) at pH 8.0. This was followed by the addition of 20 µL of OPA (1 mg/mL in methanol) and incubation in the dark at room temperature for 30 min to allow for the formation of a fluorometrically detectable GSH isoindole fluorophore. Finally, GSH standards or samples were injected into the HPLC system and allowed to run for 15 min to ensure all unidentified substances eluted from the column before subsequent injections.

**Figure 1:**
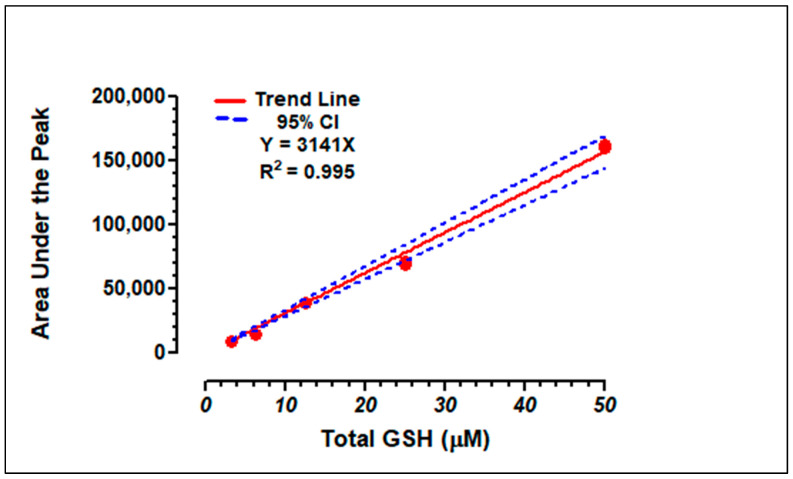
Linear regression of total GSH standard curve. The red dots represent triplicate values for injected standards of total GSH at 50, 25, 12.5, 6.25, and 3.125 µM concentrations. The blue dashed lines represent the 95% confidence intervals of the linear regression.

**Figure 2:**
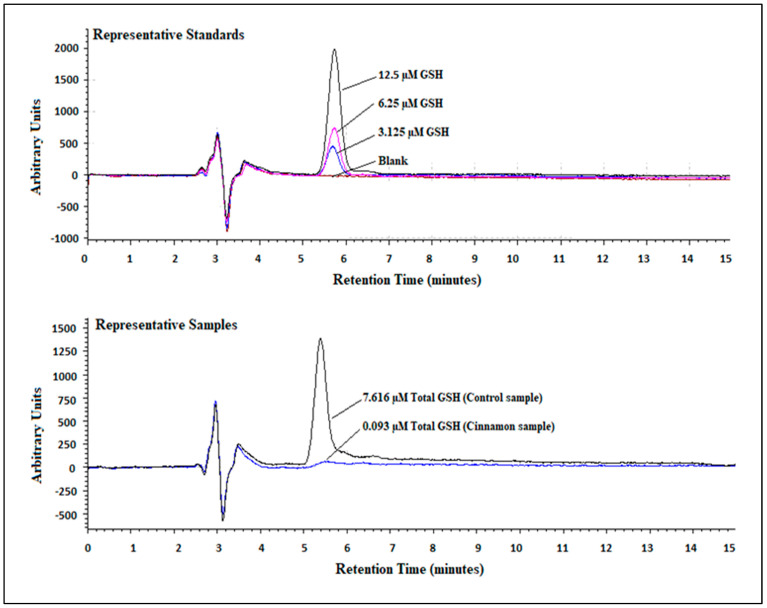
Tracings of representative standard (top) and sample (bottom) chromatograms. Representative sample chromatograms were chosen to illustrate the range of total GSH concentrations in OKF6/TERT-2 cells treated with E-liquids ± flavors. The blank contained 0.00 µM GSH. Peaks were integrated and quantified fluorometrically using excitation and emission wavelengths set at 350 and 420, respectively. All total GSH samples analyzed achieved levels above the limit of detection (LOD) and limit of quantitation (LOQ) and ranged from 0.033 and 22.961 µM.

Three sets of GSH standard curves were generated and found to be linear (R^2^ = 0.999). Retention times for GSH ranged between 5.78 and 5.87 min. Figure 1 shows the linear regression for GSH, and Table 1 details the standard curve data. Figure 2 illustrates representative standards and sample chromatograms.

### 2.10. Mucins and Tight Junction Gene Expression

OKF6/TERT-2 cells were grown and treated with 1% E-liquids as above for 24 h. Supernatant was stored for released glycoprotein evaluation. Confluent monolayers were washed once with 1 mL PBS, and total RNA was extracted using the mirVana miRNA isolation kit, phenol/chloroform, and 100% ethanol, as described by the manufacturer’s instructions. The NanoDrop 2000c (Thermo Fisher Scientific, Waltham, MA, USA) was used to determine RNA concentrations, and cDNA was obtained with the VILO reverse transcription kit. TaqMan assays for *18S rRNA*, *muc1*, *muc4*, *muc5b*, *occludin*, *claudin-1*, and *ZO-1* were used in the amplification and detection of these genes. The QuantStudio 3 qPCR cycler (Applied Biosystems, Waltham, MA, USA) was used to determine cycle threshold (Ct) values. Denaturation and polymerase activation was performed by incubation at 95 °C for 2 min. Once activation was completed, cDNA was amplified in 50 cycles: denaturation at 95 °C for 10 s and 60 °C for 30 s for annealing and extension. The data were calculated using the 2^−ΔΔCt^ method where the *18S rRNA* served as the housekeeping control.

### 2.11. Released Glycoprotein Concentration and SDS-Page

OKF6/TERT-2 cells were grown and treated with 1% E-liquids as above. Supernatants were collected 24 h post-E-liquid treatments. Using Amicon Ultra 15 centrifugal filters with a 10 kDa cutoff, 10 mL of each sample was concentrated by centrifugation at 6500× *g* to obtain about 500 μL retentates from each treatment. To quantify the amount of protein in each concentrated sample, the nanodrop was used on the ProteinA280 BSA settings. After determining protein concentrations, 50 μg of protein from each sample, plus 2.5 mM DTT and loading buffer, was mixed in a final volume of 40 μL. Equal amounts of fresh DFK media or human saliva were loaded as negative and positive controls, respectively. Proteins were separated using Genscript 4–20% gradient gels (GenScript Biotech, Piscataway, NJ, USA) via the sodium dodecyl sulfate polyacrylamide gel electrophoresis (SDS-PAGE) technique. The gels were run for 3.5 h to allow the high molecular weight glycoproteins to enter the gel. The gels were then fixed overnight in 15% trichloroacetic acid. Alcian Blue staining was used to visualize glycoproteins only, following previously established methods [30]. Gels were photographed using a Protein Simple gel imager (San Jose, CA, USA). ImageJ 1.53t was used to quantify the glycoprotein bands on each SDS-PAGE run. The signal intensity was measured, and the average intensity for each treatment was plotted and normalized to the control.

### 2.12. ELISA Determination of TNFα, IL-6, and IL-8

Supernatants from 1% E-liquid-treated and control OKF6/TERT-2 cells were collected and stored at −80 °C. Enzyme-linked immunosorbent assays (ELISA) were used to quantify TNFα, IL-6, and IL-8 production. ELISA kits were purchased from Invitrogen, through Thermo Fisher Scientific (Waltham, MA, USA). Assays were performed in 96-well plates according to manufacturer’s instructions, and absorbances read at wavelengths of 450 nm and 570 nm using a Synergy H1 (Bioteck, Winooski, VT, USA) microplate reader. Results were calculated to pg/mL based on the assay’s standard curve and normalized to the average number of live OKF6/TERT-2 cells per well.

### 2.13. Statistical Analysis

For each treatment group in all the experiments, the means ± standard error of the means (SEM) were calculated. Statistical significance between treatment groups for all experiments was determined using one-way ANOVA, followed by Bonferroni post hoc analysis, except for the wound healing experiment, which utilized a two-way ANOVA, followed by Bonferroni post hoc analysis. Differences were considered statistically significant when *p* < 0.05.

## 3. Results

### 3.1. OKF6/TERT-2 Cell Morphology

**Figure 3:**
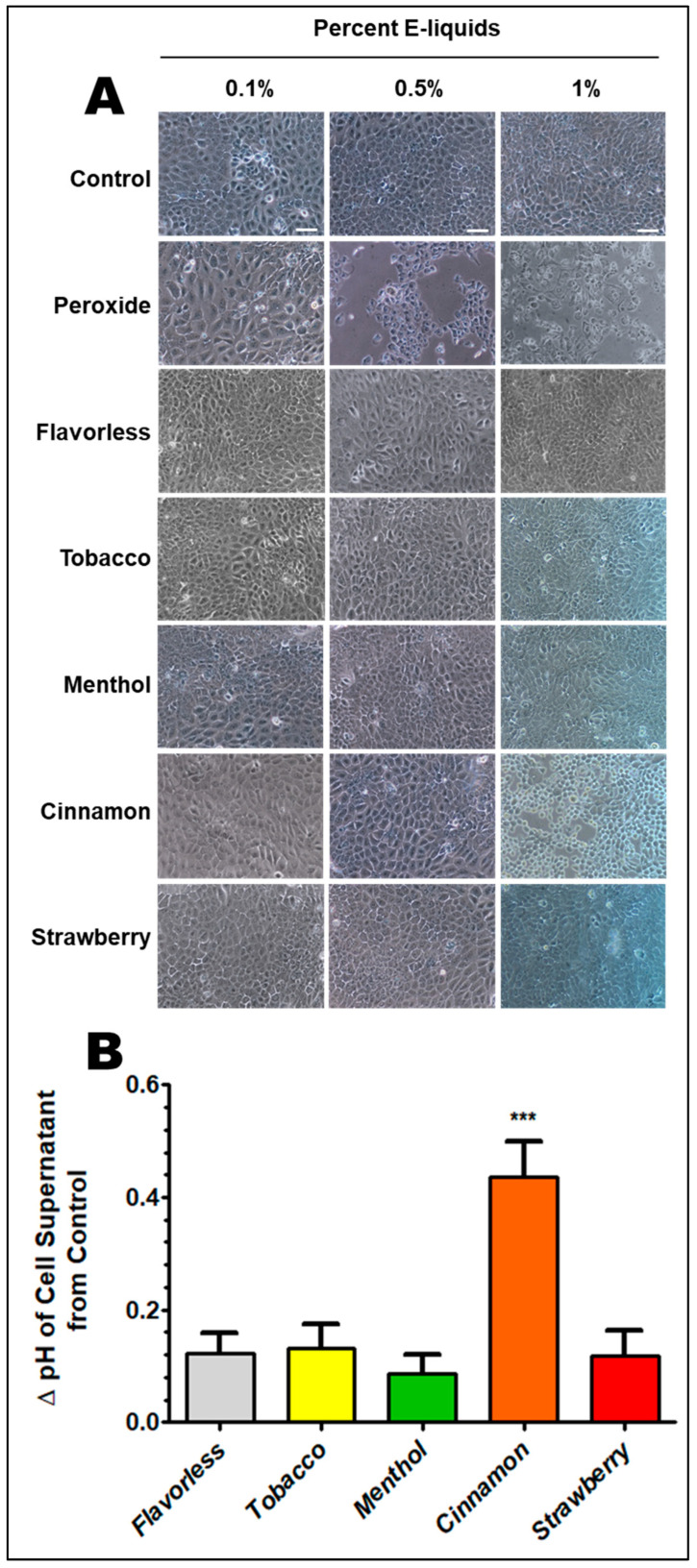
Micrographs of OKF6/TERT-2 cell monolayers > 90% confluent treated with peroxide or E-liquids ± flavors in DFK media for 24 h at standard conditions (**A**). Magnification = 100×. White bars represent 100 μm for all micrographs. Each micrograph is a representative image from four independent experiments. Change in supernatant pH from control cultures after one day of E-liquid exposure on OKF6/TERT-2 cells (**B**). Each bar represents the average change in pH between each flavor and the control, n = 7. Error bars represent the standard error of the mean. *** *p* < 0.001.

Figure 3A illustrates the typical morphology of confluent OKF6/TERT-2 cells grown in DFK medium (untreated control, row 1). At confluency, the monolayer of cells shows a conspicuous cobblestone appearance. After treatment with 0.1% hydrogen peroxide for 24 h, cells begin to lose their normal morphology and demonstrate reduced surface area coverage, as expected. The cells appear flatter and are thinly dispersed over the surface of the wells. When cultured with 0.5% and 1% hydrogen peroxide for 24 h, cells begin to detach from the surface, leaving open areas; a sign of cell death, as expected. Treatments with 0.1% and 0.5% E-liquid ± flavors for 24 h do not significantly alter the morphology and confluency of the cells. Increasing the E-liquids ± tobacco, menthol, or strawberry to 1% does not appear to affect cellular confluency and/or morphology. In contrast, 1% cinnamon-flavored E-liquid treatment for 24 h severely impacts cellular confluency and morphology; the healthy cobblestone appearance is lost. Since 0.1% and 0.5% E-liquids have minimal to no apparent effect on OKF6/TERT-2 cells, the remaining experiments in this report are conducted using only the 1% E-liquids. Figure 3B shows the change in pH of OKF6/TERT-2 cell supernatants after one-day cultures with and without 1% E-liquids ± flavors. For all treatments, the change in pH is less than 0.2 units, except for cultures treated with cinnamon E-liquid, where the change is above 0.4 units. Average pH measurements for every group ranged between 7.21 and 7.68. The only flavor that yields a significant difference in pH from the control is the cinnamon flavor (*p* < 0.001). All other conditions yield no significant differences from the control. 

### 3.2. Wound Healing Assay

**Figure 4:**
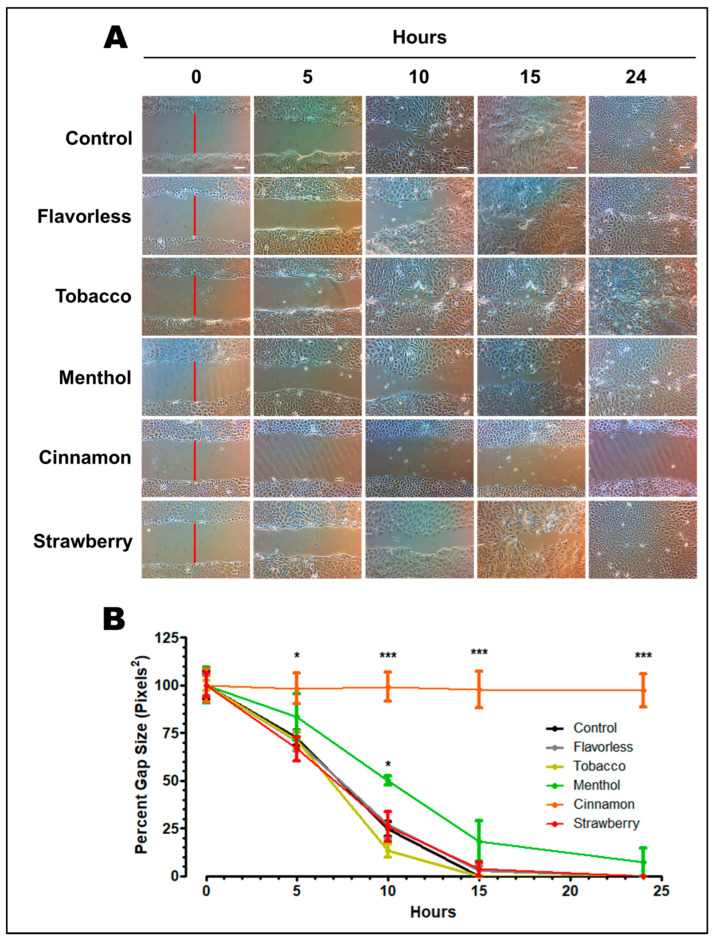
Micrographs of OKF6/TERT-2 cell monolayers grown to > 90% confluent and scratched using a 1 mL pipet tip across each well diameter (**A**). Representative images from two independent experiments. Magnification = 100×. White bars represent 100 μm for all micrographs. Vertical red lines indicate the initial wound gap. Wound gap size was measured over time across all conditions (**B**). Quantification was performed via ImageJ. Each time point represents the mean ± SEM (n = 6) of the gap size as a percentage of the initial gap size. * *p* < 0.05; *** *p* < 0.001.

The oral mucosa prevents microbes and microbial products as well as other environmental assaults from infiltrating deeper tissues, especially following oral surgical procedures. Therefore, it is paramount that oral epithelial cells repair any wounds to prevent further tissue damage from occurring. To test this repair function, OKF6/TERT-2 cell monolayers were mechanically injured and allowed to recover in the presence of 1% E-liquids ± flavors. Qualitatively (Figure 4A) and quantitatively (Figure 4B), control cells repair the wound by 15 h post-injury, as expected [30]. Cells treated with 1% E-liquids ± tobacco or strawberry recover like the control. Cells treated with 1% menthol-flavored E-liquid show a slight delay in recovery, which is significant at 10 h (*p* < 0.05). However, cells treated with cinnamon-flavored E-liquid show no post-injury recovery throughout the length of the experiment (Figure 4). In addition, cinnamon-treated cells lose their cobblestone appearance and morphology by 24 h (Figure 4A), confirming the results in Figure 3A. Overall, experimental results from the wound healing assay demonstrate a flavor-dependent effect upon OKF6/TERT-2 cell wound recovery rate and wound closure.

### 3.3. Oxidative Stress as Indexed by Total GSH

**Figure 5:**
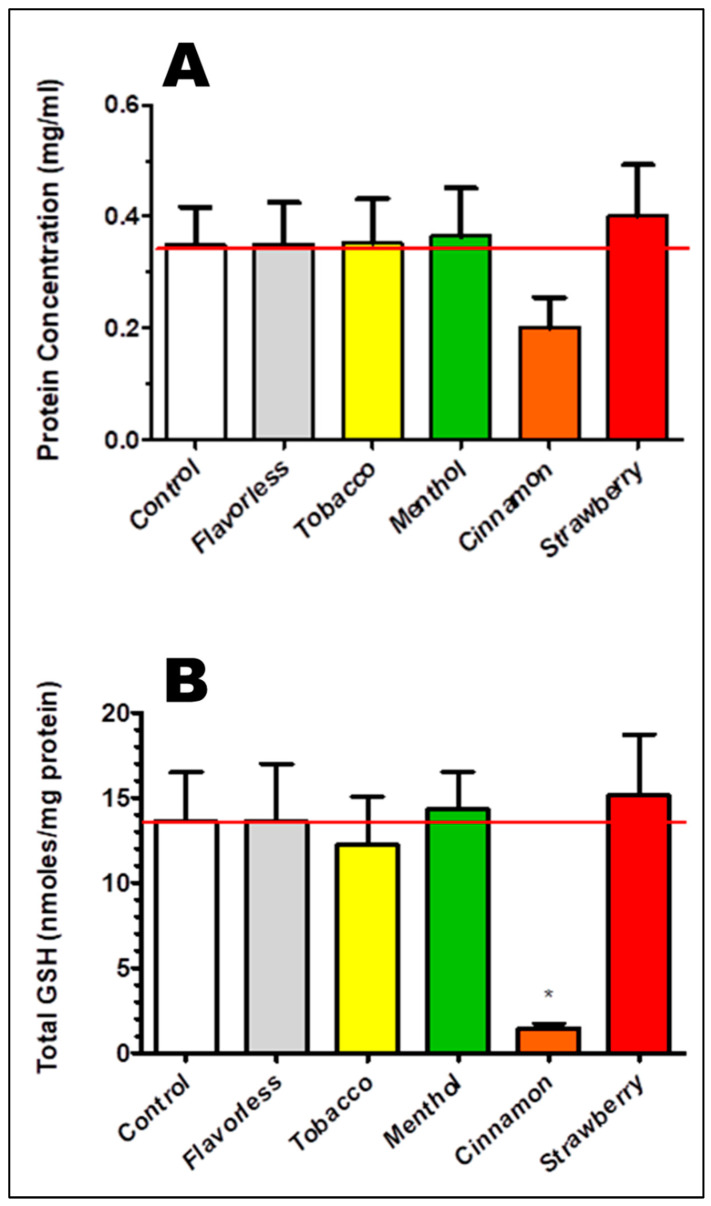
Protein content (**A**) and total GSH concentrations (**B**) from OKF6/TERT-2 cell cultures following 1% E-liquid ± flavors treatments for 24 h. Each bar indicates mean ± SEM (n = 10). Red lines represent the mean of control. * *p* < 0.05.

As an index of oxidative stress, total GSH was measured from OKF6/TERT-2 cells treated with 1% E-liquid ± flavors for 24 h. Pellet-derived filtrates of cultured OKF6/TERT-2 cells were used to determine concentrations of total GSH as a function of total protein. Figure 5A shows that protein concentrations from OKF6/TERT-2 cells with E-liquid treatments are comparable to the control, although protein levels trend lower for cells treated with cinnamon-flavored E-liquid. Figure 5B illustrates the concentrations of total GSH following treatment with 1% E-liquid ± flavors for 24 h. Control OKF6/TERT-2 monolayers yield an average of 13.64 ± 2.85 nanomoles/mg protein per total GSH. Except for the cells treated with cinnamon, which are significantly lower than the control (*p* < 0.05), E-liquid ± all other flavors has no effect on total GSH production as compared to the control. This suggests that cinnamon-flavored E-liquid treatment on OKF6/TERT-2 cells leads to the loss of total GSH. Overall, these results, along with Figure 3A, indicate that the cells are dead or dying, which is supported by Ka et al. (2003) [42] as well as our previous study [30]. Thus, the majority of total GSH is lost in the supernatant [81].

### 3.4. Gene Expression of Mucins and Tight Junction Genes

**Figure 6:**
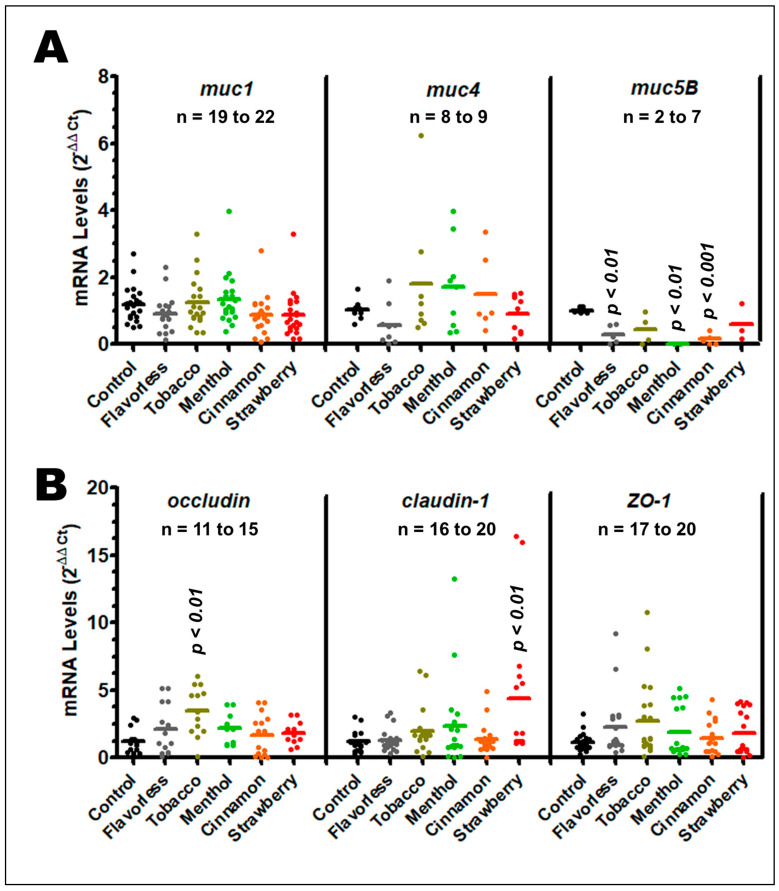
Gene expression levels of mucins *muc1*, *muc4*, and *muc5B* (**A**) and tight junction genes *occludin*, *claudin-1*, and *ZO-1* (**B**) after exposure to 1% E-liquids ± flavors for 24 h and quantified by qPCR. Means are indicated by horizontal lines, and values are represented by colored dots. Significant differences compared to the control are indicated by *p* values. Multiple Ct values were undetectable across samples and experiments. In addition, any ΔΔCt value that was outside of 2× standard deviations was considered an outlier and not used in the analysis.

To further assess the physiological function of OKF6/TERT-2 cells, mucins and tight junctions were evaluated under treatments with 1% E-liquids ± flavors for 24 h. Mucins play a crucial role in the homeostasis of the oral epithelia by maintaining hydration and lubrication and minimize harm during host-microbe interactions. Tight junctions help prevent exogenous materials from leaking through the epithelium into connective tissues and maintain the morphology and integrity of the cells. Figure 6 shows the expression levels of mucins (A) and tight junction genes (B). Treatments with 1% E-liquids ± flavors for 24 h yield no effect on *muc1*, *muc4*, and *ZO-1.* However, there is a significant downregulation of *muc5b* when cells are exposed to flavorless, cinnamon, and menthol E-liquids (*p* < 0.01). It is important to note that many Ct values for *muc5b* were undetermined because they fell below the threshold of detection in the qPCR instrument, suggesting even lower levels of expression. In addition, there is an upregulation of *occludin* and *claudin-1* when cells are exposed to tobacco and strawberry E-liquids (*p* < 0.01), respectively.

### 3.5. Released Glycoproteins

**Figure 7:**
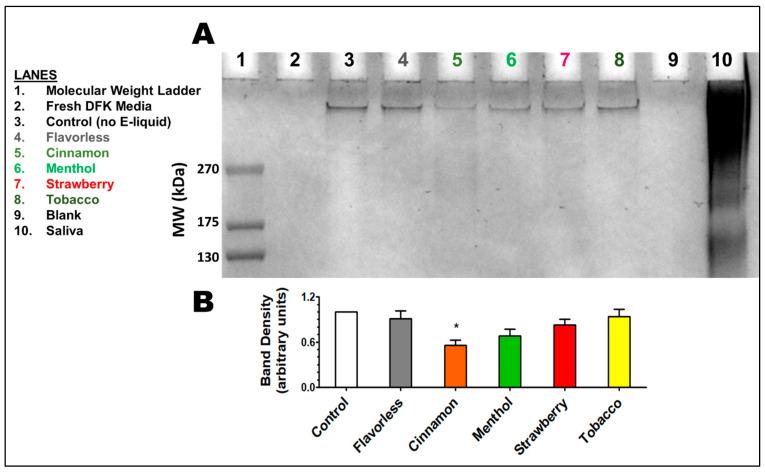
High molecular weight glycoproteins released by OKF6 cells. Alcian Blue-stained SDS-PAGE showing high molecular weight glycoproteins in OKF6/TER-2 supernatants after treatment with E-liquids ± flavors (**A**). Each lane contains 50 µg of protein. Representative gel of four separate experiments. Quantification of band density using ImageJ 1.53t (**B**). Bars represent means ± SEM (n = 4). * *p* < 0.05 compared to the control. The colored numbers on the gel (**A**) correspond to the colored numbers in the lane legend to the left.

The oral epithelium is constantly exposed to microbial and environmental assaults. Membrane-bound glycoproteins, such as mucins, are a part of the protective mechanism to help alleviate these burdens by continuously being released from the cellular surface. Glycoprotein release from OKF6/TERT-2 cells upon treatment with 1% E-liquids ± flavors for 24 h was evaluated by SDS-PAGE. In Figure 7A, the bands corresponding to glycoproteins found in the supernatant show a high molecular weight (MW), indicating these are heavily glycosylated proteins, most likely mucins. The glycoprotein bands after E-liquid treatments (lanes 4, 6, 7, and 8) display a slight decrease in band intensity compared to the control band (lane 3). This is supported by quantification from four separate experiments (Figure 7B) showing a decrease in band density. The low density of the glycoprotein band in lane 5, corresponding to the cinnamon-flavored E-liquid treatment (Figure 7A), indicates a much lower abundance of glycoprotein secretion. Quantification of this band (Figure 7B) shows a significant decrease compared to the control (*p* < 0.05). Our results indicate that the secretion of this high MW glycoprotein by OKF6/TERT-2 cells is significantly decreased upon exposure to cinnamon E-liquids.

### 3.6. Imflammatory Response as Indexed by IL-6 and IL-8

**Figure 8:**
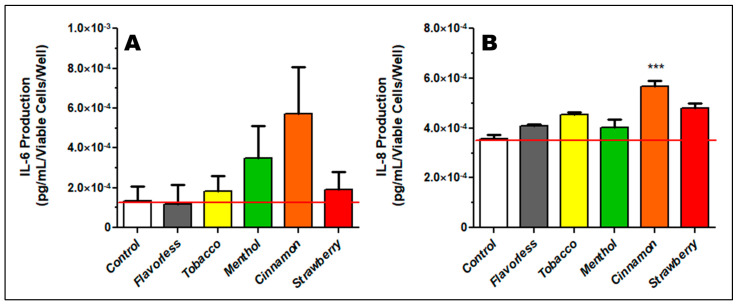
Concentrations of IL-6 (**A**) and IL-8 (**B**) from the supernatants of OKF6/TERT-2 cell cultures treated with 1% E-liquid ± flavors for 24 h. Each bar indicates mean ± SEM for IL-6 (n = 6) and for IL-8 (n = 8) per viable cell. *** *p* < 0.001. Red lines represent the mean of controls.

Cytokine release by oral epithelial cells is a sign of inflammation in the mucosa. To determine whether oral epithelial cells respond to E-liquid ± flavors by producing pro-inflammatory cytokines, ELISA was performed on the supernatants. To this end, supernatants of cultured OKF6/TERT-2 cells were used to determine the production of IL-6 and IL-8 as a function of viable cells/well. The average numbers of cells/well, published elsewhere [30], are as follows: control 574,341; flavorless 469,718; tobacco 491,353; menthol 367,165; cinnamon 173,859; and strawberry 378,150. Figure 8 illustrates the production of IL-6 and IL-8 following treatment with 1% E-liquid ± flavors for 24 h. E-liquids ± all flavors have no significant effect on IL-6 production (Figure 8A), although cinnamon treatment results in an upward trend. As seen in Figure 8B, only the cinnamon-flavored E-liquid increases IL-8 production (*p* < 0.05). These results suggest that cinnamon-flavored E-liquids may induce an inflammatory response on OKF6/TERT-2 cells. All TNFα measurements were undetectable using this ELISA kit.

## 4. Discussion

In this study, we demonstrated that exposure to certain flavored E-liquids results in pathophysiological effects to the OKF6/TERT-2 in vitro model of oral epithelial cells. This study is among the first to show the multifaceted effects of E-liquids on oral biology by means of investigating cell morphology, wound healing capabilities, total GSH, gene expression of mucins and tight junctions, glycoprotein release, and pro-inflammatory cytokine production. The intent of this investigation is not to determine exhaustive dose-dependent effects on all possible flavors of E-liquids. Rather, its intent is to determine whether these E-liquids have pathophysiological effects on oral keratinocytes.

OKF6/TERT-2 cellular morphology deteriorates at a concentration of 1% flavored E-liquid but not at lower doses. Therefore, 1% E-liquid ± flavors were selected as the treatment concentration for all remaining experiments. Our previous study indicates that 1% unflavored E-liquid + nicotine treatment does not induce cytotoxic effects on OKF6/TERT cells, which is in agreement with most other studies on oral epithelial cells and other cell lines [30,82,83]. However, one study found that cinnamon-flavored E-liquids have previously been found to decrease cell viability of A549 cells with nicotine, but this detrimental effect was mitigated without nicotine [84]. The control samples in this study with unflavored E-liquid containing nicotine do not result in increased oxidative stress, pro-inflammatory cytokine production, or decreased wound healing capabilities. Interestingly, cell cultures with cinnamon-flavored E-liquid result in a significant pH increase, not seen with other flavors. Cinnamaldehyde is a naturally occurring compound found in cinnamon bark, and it is a constituent of E-liquid cinnamon flavoring. This compound has been previously investigated as a cytotoxic compound to human embryonic and lung cells, and it is known to be a skin and eye irritant [85,86]. In contrast, the benefits of cinnamaldehyde are well documented, to include anti-inflammatory, antimicrobial, and antitumor effects as well as therapeutic effects on diabetes and cardiovascular disease [87]. These differences may be due to route of administration, concentration administered, and/or types of cells implicated. While cinnamaldehyde, being a weak base, could explain the alkaline pH, a metabolic mechanism causing ion imbalances might be responsible for the significant pH increase. In clinical terms, alkaline pH is not generally considered a pathological state; rather, it may be considered protective against the various harms of acidity in the oral cavity, including cariogenesis [88].

The wound healing assay is a metric which evaluates both viability and cell migration, both characteristics which are critical to the integrity of mucosal surfaces in the oral cavity in order to maintain its primary function as a barrier. Cinnamon- and, to a lesser extent, menthol-flavored E-liquids disrupt OKF6/TERT-2 wound healing capabilities. Shaikh et al. (2019) demonstrated that a range of unflavored E-liquids between 0.1% and 10% cause a dose-dependent decrease in wound healing time of OKF6 oral epithelial cells; however, tests of significance were not employed [89]. Our study investigated wound healing in a time-dependent manner through measurement of gap size over time, rather than quantifying the time required to re-establish confluency. Our study did not find a disruption in wound healing when treated with unflavored E-liquids, which is in contrast to Shaikh et al. (2019) who found a decrease in OKF6 wound healing time at 1%, 5%, and 10% of E-liquid [89]. Wound healing of mucosal surfaces follows four stages: hemostasis, inflammation, proliferation, and remodeling [90]. Disruption to the wound healing properties of the oral epithelium is likely to impact one or more of these stages, given that the cascade of wound healing cannot be completed without adequate re-epithelialization properties of keratinocytes, such as the OKF6 model used in this study. Previous studies have found ECIG device use to be associated with increased postoperative surgical complications [91]. Our study demonstrates on a microscopic level that certain flavors such as cinnamon and menthol may be implicated in such post-surgical complications, particularly in the oral cavity. Vasoconstrictive effects of nicotine are commonly cited as a causative factor of poor healing after oral surgical procedures; however, our study indicates that flavoring compounds from ECIGs may be an additional independent factor further exacerbating healing complications.

MUC5B plays a predominant and crucial role on water retention in saliva, viscoelasticity, moistening, and lubrication [92,93]. Three of the five E-liquids tested significantly downregulate *muc5b* expression in OKF6/TERT-2 cells, but no alterations in *muc1* and *muc4* were noted. Such results could be affected by timing, where different mRNA levels could be read at different time points in the experiment. For example, *muc5b* could be upregulated within the first 10 h of treatment and downregulated by 24 h. Low levels of *muc5b* expression are highly correlated with xerostomia [94], and vaping is associated with this anomaly [95], thereby establishing a correlation between vaping and xerostomia at the molecular level. In the lower respiratory mucosa, cigarette smoking increases production of mucins MUC1 and MUC4 in bronchial epithelial cells [96]. Although there are no reports on the expression of these mucins on oral epithelial cells, Go et al. (2020) reported that in vitro treatment of human middle ear epithelial cells with tobacco and menthol-flavored E-liquids results in increased MUC4 expression; however, cinnamon was not investigated in their study [97]. Physiologically, mucins play a role in growth, differentiation, and signaling of cells, and their function depends on whether the mucins are membrane-bound or soluble. Increased aberrant expression of MUC4 has been shown in multiple human cancers, with evidence that cancer cells may use mucins for cell proliferation, survival, and protection against immune defenses [98]. Recently, Kohli et al. (2019) showed that MUC4 expression is also increased in oral squamous cell carcinoma tumors and may play a role in oral carcinogenesis [99].

Occludin is a protein that facilitates cohesion of epithelial tight junctions, which is a critical function of the oral epithelium. Similar to occludin, claudin-1 plays a role in formation of the physical oral barrier and paracellular transport, and also interacts with occludin to form junction complexes [71,100]. In a mouse model, radiation treatment for head and neck cancer often results in upregulation of *occludin*; which might be complicated by oral mucositis [101]. Alarmingly, overexpression of *claudin-1* is correlated with oral squamous cell carcinoma [102,103]. Various oral lesions such as nicotine stomatitis (also known as smoker’s palate), hairy tongue, and angular cheilitis may be seen after prolonged ECIG use [104]. In our study, tobacco and strawberry flavorings significantly increase the expression of *occludin* and *claudin-1*, respectively. We posit that increased expression of *occludin* and *claudin-1* following treatment with tobacco- and strawberry-flavored E-liquids may result in oral pathologies as indicated above. Thus, the roles of *occludin* and *claudin-1* expression in these clinical manifestations of ECIG use should be further investigated. In contrast, decreased oral mucosal *occludin* gene expression has also been associated with various pathological states, including increased severity of oral lichen planus [105]. Furthermore, decreased tissue expression of *occludin* has been linked with various intestinal permeability disorders [106]. Interestingly, exposure of airway epithelial cells to ECIG aerosol dissolved in culture media results in disruption of tight junctions, although the total amount of protein remains constant [107]. The overexpression noted in our study could be a response of the damage in tight junctions as seen by Raduka et al. (2023) [107]. To our knowledge, no other studies have investigated expression of *occludin* and *claudin-1* in the oral epithelium after exposure to flavored or unflavored E-liquids.

MUC7 and MUC5B are commonly considered to be the major salivary glycoproteins and comprise a significant portion of saliva, approximately 20% of the total protein present [108]. They have various functions, some of which include as lubricants, barriers to desiccation [109], and agglutination with bacteria to facilitate clearance from the oral cavity [110]. Our study indicates that mucin secretion from OKF6/TERT-2 cells is significantly decreased when treated with cinnamon-flavored E-liquids. It has been previously reported that MUC5B is a highly abundant mucin in human saliva, with a molecular weight in the thousands of kilodaltons, barely entering the top of an SDS-PAGE gel [111,112,113,114]. In addition, MUC5B is more resistant to degradation than other salivary glycoproteins [113]. Based on the downregulation of *muc5B* mRNA and its correlation with the decrease in high MW glycoproteins after E-liquid treatments, we speculate that the bands seen in SDS-PAGE correspond to MUC5B. Overall, the data indicate that some ECIG flavors may result in the disruption of salivary mucin composition, which could lead to clinical manifestations such as xerostomia, oral microbial dysbiosis, periodontal disease, and others [95,115,116].

In this study, cinnamon has the propensity to induce oxidative and inflammatory responses in OKF6/TERT-2 oral epithelial cells. Unflavored E-liquid does not have any pro-inflammatory or oxidative effects on the cells. However, other studies have determined that aerosolized unflavored E-liquid dose-dependently decreases intracellular levels of glutathione in normal human oral keratinocytes [66]. Lee et al. [117] found that 0.3% and 1% cinnamon treatments induce a 3- and 6-fold increase in ROS production in induced pluripotent stem cells (iPSCs), respectively. Similarly, 1% menthol treatment induces a 10-fold increase in ROS production in iPSCs [117]. Cinnamon flavoring has been previously shown to be harmful due to the presence of cinnamaldehyde [118]. The effects of aldehydes from cigarette smoke have been well established as having physiological consequences; however, recent studies also confirm that cinnamaldehyde impairs human bronchial epithelial cells’ mitochondrial function and ciliary beat frequency, thereby increasing the risk of respiratory infections [118]. Cinnamaldehyde has also been implicated in impaired respiratory immune cell function [119] and neutrophil phagocytic capabilities [120].

Generation of intracellular GSH is a means that allows cells to cope with oxidative stress and the presence of ROS. In the event of oxidative stress, the activity of γ-glutamyl cysteine synthase, and hence the amount of GSH, is known to increase [64,65], ultimately leading to oxidation of GSH. The oxidation of GSH neutralizes the presence of ROS by forming disulfides, either with itself to yield GSSG, or with other sulfur-containing proteins or peptides [62]. Consequently, during oxidative stress, total GSH (i.e., GSH and GSSG) increases [61,63], although GSSG contributes far less to the total compared to GSH. During homeostatic conditions, the intracellular ratio of GSSG/GSH ranges between 0.01 and 0.33 [65,121,122,123,124,125,126,127,128] but increases in response to oxidative stress. Kaushik et al. (2008) found that 50 µg/mL of cigarette smoke condensate significantly increased the production of ROS and the activity of γ-glutamyl cysteine synthase, and nearly doubled the concentrations of both GSH and GSSG in human lung epithelial type-II cells (A549) [125]. In contrast, the GSSG/GSH ratio only increased from 0.07 in controls to 0.10 after treatment with the cigarette smoke condensate. Similarly, Li et al. (2002) reported that exposure of human bronchial epithelial cells (BEAS-2B) to diesel exhaust particles stimulated ROS production and increased the GSSG/GSH ratio from approximately 0.02 to 0.33; however, they make no mention of the actual GSSG or GSH levels from which this ratio was derived [126]. With this said, care needs to be taken in the determination of the GSSG/GSH ratio since intracellular GSH levels can be as much as 100 times greater than GSSG levels [122] and could ultimately lead to large discrepancies in the ratio between experiments. We used HPLC with fluorescence detection to quantitate total GSH, which falls within the GSH range of other studies [65,121,122,123,124,125,126,127,128]. The variations noted in GSH levels between studies could be attributed to the detection method used to measure GSH or to the cell type investigated.

Exposure to cinnamon-flavored E-liquid significantly reduces intracellular total GSH. GSH depletion is correlated with apoptosis [129]. This suggests that cinnamon-flavored E-liquid induces a cytotoxic oxidative event resulting in apoptosis, as found by others [39,66,67,130]. These results and the results of our previous study [30] indicate that cinnamon-flavored E-liquid is able to compromise cell viability and induce cell death, most likely due to excessive oxidative stress and apoptosis. Although not significant, the protein content of OKF6/TERT-2 cells exposed to cinnamon-flavored E-liquid is conspicuously lower than the control and the other E-liquid ± flavor treatments, and could be an additional sign that the cells are dead or dying. Furthermore, since proteins are much larger in comparison to GSH, less protein is lost to the supernatant during the process of cell death. This incongruous loss of GSH and protein, most likely in the form of apoptotic bodies in the supernatant, aligns well with the GSH depletion we observe in OKF6/TERT-2 cells.

Currently, very little information is available regarding the effects of E-liquids ± flavors (or their aerosols) on intracellular GSH levels either in vivo or in vitro. Presented here are the few available investigations found in the primary literature. Ji et al. (2016) [66] report that flavorless ECIG-generated aerosol decreases intracellular GSH as a result of cytotoxicity in normal human oral keratinocytes. This is in opposition to the results presented in our study. Using a human endothelial cell line (EA.hy926), Kerasioto et al. (2020) [131] report that tobacco-flavored E-liquid has no effect on GSH levels, supporting our results. However, they also report that tobacco-flavored E-liquid increases production of ROS. Unlike our results, Herbert et al. (2023) [132] show menthol-flavored E-liquid condensates to significantly deplete GSH from precision cut lung slices. Although total GSH was not measured, Muthumalage et al. (2018) [39] found ROS production to increase in two monocytic cell types (MM6 and U937) after exposure to the flavoring chemical cinnamaldehyde (an important constituent of cinnamon flavoring). The assumption here is that increased ROS production equates to increased oxidative stress, which in turn could lead to depletion of intracellular GSH if the oxidative stress were severe enough. Wavreil and Heggland (2019) [130] report a similar increase in ROS production in the human osteosarcoma cell line MG-63 exposed to cinnamon-flavored E-liquids and aerosols, while Noel and Ghosh (2022) report an increase in ROS production in BEAS-2B cells exposed to strawberry-flavored aerosol [133]. The aforementioned discrepancies noted between our results and those of others are most likely explained by differences in the methodology of E-liquid exposure (i.e., concentration of E-liquid used or puff topography), the mode of GSH detection, and the cell type used in other investigations [65,121,122,123,124,125,126,127,128].

Cinnamon-flavored E-liquid treatment results in increased production of IL-8 and an upward trend for IL-6 from oral epithelial cells. These findings suggest that cinnamon E-liquid may result in the initiation of a pro-inflammatory cascade that could lead to local immune and oxidative responses at the epithelial or mucosal level. In agreement with our data, Remenzoni et al. (2022) found that IL-1α, IL-1β, and IL-6 production by oral epithelial cells significantly increases after exposure to ECIG aerosols [134]. Similarly, human airway epithelial H292 cells display increased secretion of IL-6 and IL-8 when exposed to cinnamon E-liquid [40]. Moreover, BEAS-2B lung epithelial cells upregulate TNFα and IL-8 in response to vanilla-flavored aerosol [133]. In the same cell line, exposure to acetoin and maltol induces IL-8 production [43]. TNFα production in the OKF6/TERT-2 cell line was undetectable with our ELISA methodology. This is not surprising since a study by Zhang and coworkers (2018) [135] evaluated the production of TNFα in OKF6/TERT-2 cells and found 43.25 pg/mL of this cytokine in their control using an ultrasensitive ELISA methodology published by Diala et al. (2013) [136]. Increased pro-inflammatory cytokine production in the oral environment has been linked with oral squamous cell carcinoma through an inflammatory pathway [137,138,139]. Pro-inflammatory cytokines, through related inflammatory and dysbiotic pathways, may contribute to the development and/or progression of periodontal disease [140]. Similar systemic pro-inflammatory mechanisms are implicated in the development of atherosclerosis, hypertension, and thrombosis, among others.

The current study is not without limitations. First, this is an in vitro study carried out with an immortalized cell line, which may not fully reflect the complexity of cellular interactions and microenvironments found in living organisms. Secondly, the treatments evaluated in this investigation were restricted. For example, this study made use of E-liquids and did not extend to testing aerosols bubbled into culture media, which is a technique gaining popularity and may lead to more applicable and relevant results. In addition, due to current discrepancies in the impact that nicotine has on the role of flavored and unflavored E-liquids in inducing cytotoxicity, it would be useful to repeat the experimentation with and without nicotine to further establish the impact of nicotine. Furthermore, the diversity of ECIG flavoring compounds has been estimated to be in the thousands, suggesting that there may be wide variability in the effects that one flavoring has when formulated by different companies in different settings. Moreover, this study is narrowed to a single dose of 1% E-liquid in the culture media. Therefore, it is important for future studies to source multiple doses and flavorings from various companies to increase the external validity. Thirdly, the cellular outputs measured are also constrained. The quantification of metallothionine levels in cells treated with flavored E-liquids, by reverse transcription of mRNAs and qPCR, would also further develop our understanding of the cellular oxidative response to flavored E-liquid. In addition, all mRNA and cytokine measures were performed only after 24 h of treatment with no consideration at earlier time-points. Flow cytometry or fluorescent microscopy could be employed to quantify levels of Annexin V positive cells correlated to apoptosis. Caspase activation could also be evaluated using Western blotting. High-throughput RNA sequencing could be used to determine all changes in the transcriptome of our model oral epithelial cells. This study was conducted over an acute period. However chronic exposure must also be investigated so that the effects of continuous ECIG use can be further evaluated for its role in disease pathogenesis.

## 5. Conclusions

This study elucidates the effects of various flavored E-liquids on OKF6/TERT-2 oral epithelial cells with respect to morphology, wound healing capability, gene expression of mucins and tight junctions, glycoprotein production, oxidative stress, and inflammatory cytokine production. These data show that flavored E-liquids, notably cinnamon, lead to considerable pathophysiological effects in the OKF6/TERT-2 in vitro model, indicating the potential hazards of ECIG usage. Such effects may lead to more severe conditions in the oral cavity. Oral health is intimately connected to systemic health, and therefore it is important to further investigate the toxicological and pathologic effects of flavored ECIGs in the mouth.

## Figures and Tables

**Table 1:** Glutathione standard curve data.

Standard Curve Statistics						
Compound	Injected Concentration (µM)	Retention Time (min) *	Measured Concentration (µM) *	% Deviation from Injected Concentration	When Area Under Peak Is …	±95% Confidence Interval
Total Glutathione	3.125 6.250 12.500 25.000 50.000	5.775 ± 0.051 5.814 ± 0.049 5.821 ± 0.051 5.841 ± 0.052 5.873 ± 0.061	2.957 ± 0.056 4.667 ± 0.044 12.438 ± 0.024 22.496 ± 0.170 51.476 ± 0.150	−5.367 −25.328 −0.499 −10.015 2.952	9816 18,650 39,263 70,017 157,052 ± 665	±782 ±1487 ±3130 ±6299 ±12,519
Glutathione Straight Line Equation is Y = 3141X (line forced through zero); LOD (µM) = 0.00027; LOQ (µM) = 0.00083; R^2^ = 0.995%; % RSD = 11.162						

* = mean ± SEM where n = 3; Limit of detection (LOD) and limit of quantitation (LOQ) were determined based on the calibration curve. % RSD = relative standard deviation (i.e., coefficient of variation) of the regression line.

## Data Availability

The raw data supporting the conclusions of this article will be made available by the authors on request.
